# Principles and Practical Considerations for the Analysis of Disease-Associated Alternative Splicing Events Using the Gateway Cloning-Based Minigene Vectors pDESTsplice and pSpliceExpress

**DOI:** 10.3390/ijms22105154

**Published:** 2021-05-13

**Authors:** Elena Putscher, Michael Hecker, Brit Fitzner, Peter Lorenz, Uwe Klaus Zettl

**Affiliations:** 1 Division of Neuroimmunology, Department of Neurology, Rostock University Medical Center, Gehlsheimer Street 20, 18147 Rostock, Germany; elena.putscher@uni-rostock.de (E.P.); brit.fitzner@med.uni-rostock.de (B.F.); uwe.zettl@med.uni-rostock.de (U.K.Z.); 2 Rostock University Medical Center, Institute of Immunology, Schillingallee 70, 18057 Rostock, Germany; peter.lorenz@med.uni-rostock.de

**Keywords:** alternative splicing, RNA processing, splicing regulation, splicing reporter minigene assay, gateway cloning, pDESTsplice, pSpliceExpress

## Abstract

Splicing is an important RNA processing step. Genetic variations can alter the splicing process and thereby contribute to the development of various diseases. Alterations of the splicing pattern can be examined by gene expression analyses, by computational tools for predicting the effects of genetic variants on splicing, and by splicing reporter minigene assays for studying alternative splicing events under defined conditions. The minigene assay is based on transient transfection of cells with a vector containing a genomic region of interest cloned between two constitutive exons. Cloning can be accomplished by the use of restriction enzymes or by site-specific recombination using Gateway cloning. The vectors pDESTsplice and pSpliceExpress represent two minigene systems based on Gateway cloning, which are available through the Addgene plasmid repository. In this review, we describe the features of these two splicing reporter minigene systems. Moreover, we provide an overview of studies in which determinants of alternative splicing were investigated by using pDESTsplice or pSpliceExpress. The studies were reviewed with regard to the investigated splicing regulatory events and the experimental strategy to construct and perform a splicing reporter minigene assay. We further elaborate on how analyses on the regulation of RNA splicing offer promising prospects for gaining important insights into disease mechanisms.

## 1. Introduction

Splicing is an essential process in transferring information from the DNA level to the protein level [1]. The splicing process can be influenced by genetic variants, which may contribute to the development of diseases as a consequence [2]. Splice variants of human genes can be analyzed in a transcriptome-wide manner or specifically for individual genes. Various methods have been used for the analysis of alternative splicing events (ASE), such as next-generation sequencing (NGS), high-resolution microarrays, reverse transcriptase PCR (RT-PCR) assays, and computer-based tools to predict a potential effect of genetic variants and splicing factors on RNA processing [3]. An experimental approach under defined conditions but still relatable to the physiological state is needed to achieve an improved causal understanding of alterations in the splicing pattern of a specific gene. A particularly useful method for this purpose is the splicing reporter minigene assay, an approach to study the role of potential *cis*-regulatory elements and *trans*-acting factors involved in pre-mRNA splicing [3].

In this review, we describe the special features of the minigene assay and discuss how the use of the Gateway technology further facilitates this experimental procedure. We provide an overview of how the Gateway cloning-based vectors pDESTsplice and pSpliceExpress, originally developed by Kishore et al. [4], were used in the literature to obtain a better understanding of the physiological and pathological regulation of mRNA processing.

## 2. Splicing and Types of Alternative Splicing Events of Protein-Coding Genes

Splicing describes a physiological process in the cell nucleus, in which a precursor transcript is processed into a mature form. Most transcribed genes in eukaryotes consist of small sequences, called exons (for expressed regions), that are interrupted by intervening usually longer sequences, called introns (for intragenic regions). On average, a human protein-coding gene contains 11 exons and 10 introns, with an average length of 309 bps and 6355 bps, respectively [5]. During splicing, the introns are cut out and the remaining exons are joined together to form the mature RNA molecule. In the case of a protein-coding gene, the splicing of the precursor transcript (pre-mRNA) results in a messenger RNA (mRNA) [1].

**Figure 1:**
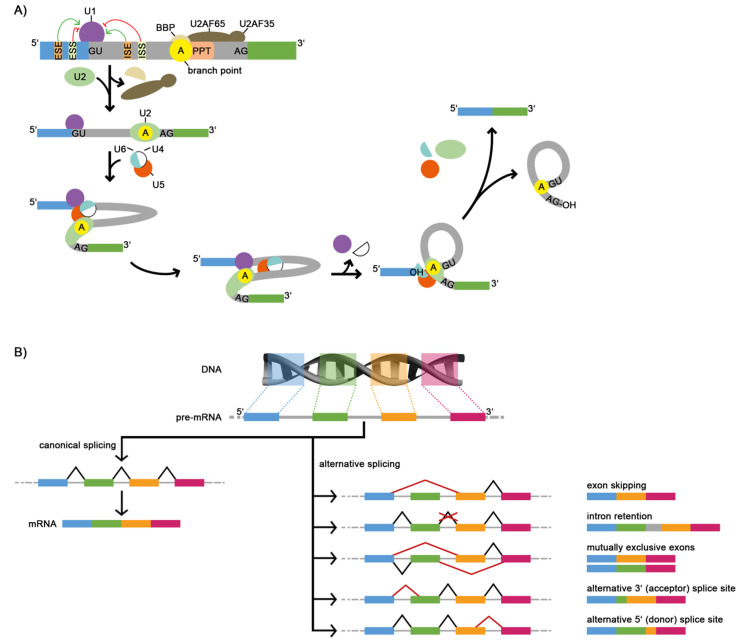
The splicing process and common types of alternative splicing events (ASE). (**A**) Simplified scheme of pre-mRNA splicing with two exons (blue and green boxes) and one intron (gray box). The *cis*-regulatory elements, namely, 5′ and 3′ splice sites, which are evolutionarily highly conserved (usually GU and AG, respectively), branch point (yellow circle), polypyrimidine tract (light pink box), and splicing enhancers and silencers (ESE, ESS, ISE, ISS, light orange and light-yellow boxes) assist the spliceosome in recognizing the 5′- and 3′-ends of the intron. Positive regulation is indicated by green arrows, while negative regulation is shown in red. The formation of the spliceosome complex leads to conformational changes of the pre-mRNA. In the first step, the U1 snRNP binds to the GU sequence at the 5′ splice site. At the same time, the branch point is bound by the branch point-binding protein (BBP) and the polypyrimidine tract is bound by U2AF. In the next step, the BBP is replaced from the branch point by the U2 snRNP. The interaction of the branch point with U2 leads to the recruitment of the U4/U5/U6 snRNP complex and thereby to the formation of the pre-catalytic spliceosome. The following change of the spliceosome conformation leads to the release of U1 and U4. Then, the interaction of U6 with U2 results in a transesterification, where the guanosine of the 5′ splice site is bound to the adenosine in the branch point. In a second transesterification step, the exons are joined together. The spliced-out intron (lariat structure) is degraded, and the U2, U5, and U6 snRNPs are released to catalyze the following splicing process. (**B**) The canonical (left) and alternative (right) splicing paths with corresponding alternative splicing events that can be distinguished. The blue-, green-, orange-, and pink-colored boxes represent 4 different exons in the 5′ to 3′ direction, while the gray lines in between represent introns. The constitutive path of intron removal (black lines) and alternative paths (red lines) are indicated. ASE: alternative splicing events, BBP: branch point-binding protein, ESE: exonic splicing enhancer, ESS: exonic splicing silencer, ISE: intronic splicing enhancer, ISS: intronic splicing silencer, PPT: polypyrimidine tract, snRNP: small nuclear ribonucleoprotein, U2AF: U2 auxiliary factor.

The splicing process occurs co-transcriptionally and is regulated by a number of short RNA sequence elements that interact with the components of the spliceosome [6] (Figure 1A). The spliceosome complex is composed of five small nuclear ribonucleoproteins (snRNPs) (U1, U2, U4, U5, and U6). Splicing is performed by a cycle of assembly and disassembly of the spliceosome complex. The spliceosome complex binds to the pre-mRNA and catalyzes the excision of an intronic sequence by forming a lariat structure. Important recognition sequences for the mRNA processing are the 5′ and 3′ splice sites (donor and acceptor, respectively), the branch point, and the polypyrimidine tract [7,8,9,10]. Furthermore, the splicing process is coordinated by the complex interplay of *cis*-regulatory elements and *trans*-acting factors [11]. The *cis*-regulatory elements are sequences (motifs) within the pre-mRNA that can either enhance or silence splicing. They are classified into exonic splicing enhancers (ESE), exonic splicing silencers (ESS), intronic splicing enhancers (ISE), and intronic splicing silencers (ISS). RNA-binding proteins (RBPs) represent *trans*-acting factors. Examples for prominent *trans*-acting RBPs that are important for the recruitment of the spliceosome complex are the protein families of the serine-arginine-rich proteins (e.g., SRSF11) and the heterogeneous nuclear ribonucleoproteins (hnRNPs) as well as the U2 auxiliary factor (U2AF), a heterodimer, which consists of a 35 kDa and a 65 kDa subunit [10,12].

The regulation of the splicing process by *cis*-regulatory elements and *trans*-acting factors allows for the use of different splice sites and thus leads to alternative splicing. ASE result in an altered exon usage compared to canonical splicing (Figure 1B). It may happen that an exon is excised and not inserted into the mature mRNA (exon skipping). It is also possible that an intron is not removed and remains within the mRNA (intron retention). The use of different splice sites can also cause the presence of mutually exclusive exons. In this case, only one of two possible exons occurs in the final mRNA, depending on which splice sites are used. Further possibilities of alternative splicing arise from the usage of different acceptor or donor splice sites, resulting in exons of different lengths. Due to the different mechanisms for the regulation of mRNA processing, alternative splicing allows the generation of different mRNAs from a single pre-mRNA molecule. Since the mRNA defines the amino acid sequence of the proteins, alternative splicing contributes to high protein diversity.

## 3. Relevance of Alternative Splicing in Health and Disease

Splicing of a particular gene can vary between different tissues. An example is the tissue-specific splicing of the calcitonin gene, which encodes for calcitonin in the thyroid and for calcitonin gene-related peptide in the nervous system [13]. On the other hand, genetic variants can markedly affect the splicing process. These variants can be alterations of only one base (single-nucleotide polymorphisms, SNPs) or of a short stretch of DNA (e.g., Alu insertion polymorphisms). Some polymorphisms are located in splicing regulatory elements and thus can cause aberrant splicing events. SNPs and *de novo* mutations can even lead to splice sites, which are usually not recognized by the spliceosome (cryptic splice sites) [2]. Such genetically driven alterations of the splicing pattern can lead to or increase the risk of disease development [2]. While some genetic effects are more tissue-specific, *cis*-regulatory effects on splicing are usually highly shared across tissues and cell types [14]. Thus, effects that a pathogenic splice variant may have in one tissue are likely to have a very similar impact in other tissues, and it also follows that investigations using cell lines in vitro are in general a good representation of the situation *in vivo*.

There are several diseases that are known to be promoted by SNPs that influence the splicing pattern. For instance, aberrant splicing has been implicated in various multifactorial immune-mediated diseases. We have previously reviewed studies in which altered alternative splicing has been investigated in the context of multiple sclerosis [15]. In this work, we found that the most studied gene was the interleukin-7 receptor (IL7R). A total of seven studies showed that skipping of exon 6, generating soluble IL7R isoforms, depends on the genotype of the SNP rs6897932, which is a well-established risk variant for multiple sclerosis. However, alteration of splicing has also been considered for therapeutic applications. In spinal muscular atrophy, homozygous mutations in the survival motor neuron 1 (SMN1) gene lead to a deficiency in the encoded protein SMN [16]. As SMN plays a role in the biogenesis of snRNPs and thus in mRNA processing, this deficiency has severe implications [17]. In patients suffering from spinal muscular atrophy, the loss of SMN cannot be compensated by the homologous SMN2 gene, as it only produces low levels of functional SMN due to substantial exon 7 skipping. To overcome the reduced protein concentration, researchers have tested therapies with antisense oligonucleotides. The antisense oligonucleotide nusinersen targets an ISS to promote SMN2 exon 7 inclusion, which leads to an increased expression of SMN, demonstrating that an effective therapeutic intervention in alternative mRNA splicing is possible [18,19].

## 4. Experimental and Bioinformatic Methods for Investigating Splicing Variants

Improved methods foster the examination of different splicing variants and disease-related ASE. Two important approaches for profiling the spliceo-transcriptome are high-density microarrays and high-throughput NGS [20,21]. Microarrays are based on probes made of short oligonucleotides, which are complementary to sequences of annotated transcripts [20]. In the case of modern microarrays, millions of probes capture exons and exon–exon junctions, enabling the expression analysis of all known human gene transcript isoforms. For high throughput NGS of isoforms, as defined, e.g., in Ensembl [22], different approaches and machines are available [23]. RNA sequencing is based on the principle that RNA is transcribed into complementary DNA (cDNA) by reverse transcriptase. After amplification of the cDNA, the prepared library is ready to be sequenced, which results in short or long sequence reads [21]. The analysis of the data then relies on the accurate mapping of the obtained sequence reads to an annotated reference genome. While microarrays require prior knowledge of previously defined transcript isoforms, RNA sequencing can be used to identify novel splicing patterns. However, measuring the levels of specific RNA isoforms remains challenging. Microarrays and RNA sequencing approaches are usually based on the analysis of short sequences. Since RNA isoforms share a large degree of similarity and only differ in a certain sequence segment, a probe or read can fit to more than one isoform. Thus, short sequences often cannot be unambiguously assigned to individual transcripts, which renders the isoform-specific determination of gene expression difficult and can lead to misinterpretations of the data. Therefore, experimental validation by event-specific and isoform-specific PCR assays is often useful. An event-specific PCR assay can confirm the presence of a specific ASE, and an isoform-specific PCR assay enables the analysis of the relative expression level of a particular RNA isoform. However, in either case, prior knowledge of a part of the examined sequence is required in order to design suitable primers for the amplification step.

On the other hand, various bioinformatic tools and databases are available to predict splicing-relevant pre-mRNA regions and to evaluate the possible role of genetic variants. Useful listings of such tools and databases can be found in the reviews by Ptok et al., Ohno et al., and Yi et al. [24,25,26]. The Human Splicing Finder website 3.1 offers a particularly comprehensive and continuously updated selection of algorithms [27]. Here, different sequence motifs, such as predicted donor and acceptor splice sites and branch points, can be examined in silico. A useful database-driven application is POSTAR2, which allows for the determination of SNPs located in experimentally validated RBP-binding sites [28]. The SNPs recorded within the POSTAR2 database might have an influence on the effect of RBP splicing factors and thus may alter the regulation of splicing. However, the main limitation of bioinformatic tools and databases for exploring splicing events is that many of them are no longer updated. Moreover, a large number of tools only focus on certain types of splicing events, and the algorithms generally provide too much false-positive information [29].

## 5. Splicing Reporter Minigene Assay via pDESTsplice and pSpliceExpress Vectors

Transcriptome measurements and bioinformatic analyses can be used to identify splice variants that are potentially involved in diseases or in dysregulated biological processes. To further investigate the regulation of these specific splice variants, researchers can perform splicing reporter minigene assays. This approach provides the possibility to study ASE under defined conditions by transient transfection of cell lines with a plasmid DNA, the minigene construct. By using minigenes, splice sites can be determined, ESE/ESS and ISE/ISS can be identified, the influence of a genetic variant on splicing can be evaluated, the role of specific *trans*-acting factors can be analyzed, and cell type-specific splicing regulatory mechanisms can be investigated [30].

The construction of a minigene relies on the principle that an exon of interest together with its flanking intronic sequences (here referred to as genomic fragment of interest, GFI) is cloned into the multiple cloning site of a vector. It is also possible that the GFI consists of an array of exons intervened by sections of flanking intronic sequences. The GFI is integrated within two constitutively expressed exons, which can be either derived from another gene or the up- and downstream exons of the same gene [3,30]. The generation of the GFI can be performed by gene synthesis or by PCR amplification of genomic DNA. Most *cis*-regulatory elements in intronic sequences can be found up to 400 bps from the exon boundaries, making this constraint a good starting point for the analysis of features that may affect splicing patterns [31,32,33].

A widely used cloning procedure for the insertion of the GFI is classical restriction digestion. In this process, restriction enzymes cut in the multiple cloning site of the vector, creating matching ends that fit to the GFI. During a subsequent ligation step, the vector and the GFI are assembled to form the minigene construct. Another possibility to clone the GFI into a vector is the Gateway cloning [34,35]. This cloning method utilizes the site-specific recombination system that is used by phage λ for the integration and the excision of its DNA in the *E. coli* genome. The convenient Gateway vector systems pDESTsplice and pSpliceExpress (Addgene plasmid #32484 and #32485) that have been developed by Kishore et al. [4] are of special interest, as they are used by various research groups for minigene experiments. The two vector systems were created by modifying the Exontrap vector pET01 (MoBiTec) to enable the generation of minigene constructs within one week. The resulting minigene constructs show a similar splicing pattern in comparison to Exontrap minigene constructs obtained by conventional cloning with restriction enzymes [4]. The pDESTsplice and pSpliceExpress vectors exhibit the special feature of pET01, namely, the exons 2 and 3 of rat insulin 2, which are controlled by a strong promoter and which are constitutively spliced together [4]. These exons surround an intronic sequence that has been modified for Gateway cloning. An important part of the modified sequence is the control of cell death B (ccdB) gene that is coding for a selection marker that inhibits cell division of the *E. coli* host. The ccdB toxin inhibits replication by trapping the gyrase in the gyrase–DNA complex, thereby stabilizing DNA double-strand breaks and blocking the passage of polymerases, which eventually leads to cell death [36]. The difference between the two vector systems is that a GFI is directly cloned into pSpliceExpress or it is first cloned into a Gateway donor vector, which is then recombined with pDESTsplice [4]. The generation of a minigene construct with pSpliceExpress relies on the so-called BP reaction and with pDESTsplice on the so-called LR reaction. In the following, the two cloning reactions will be explained in more detail.

**Figure 2:**
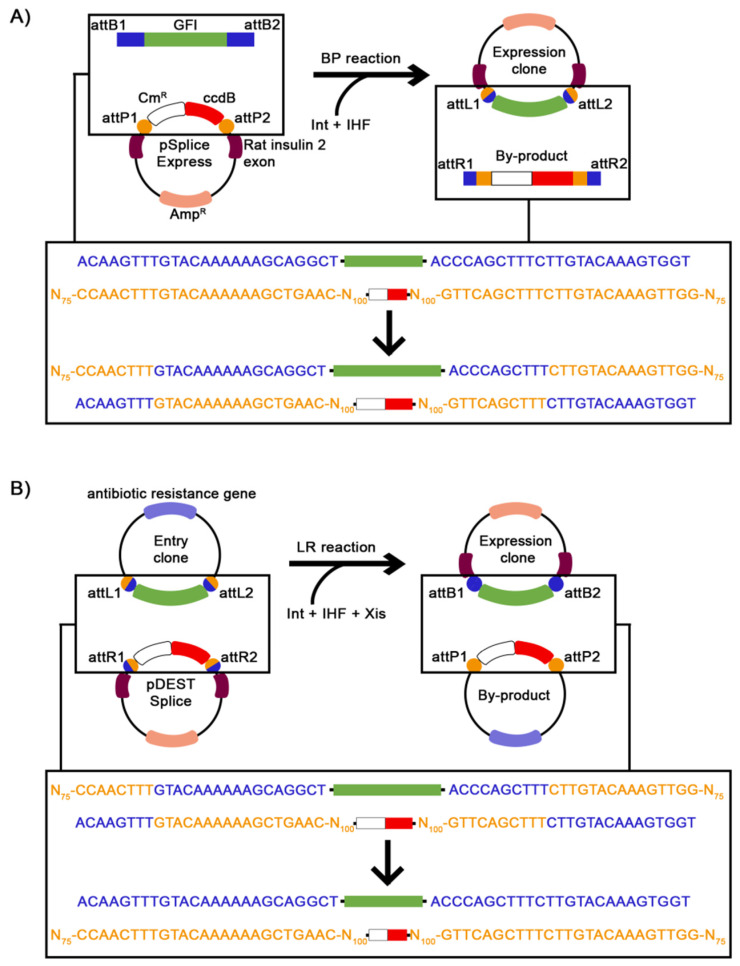
Gateway cloning with pSpliceExpress and pDESTsplice. (**A**) The BP reaction is mediated by Int and IHF and leads to the cloning of the GFI (green box) into pSpliceExpress. Initially, the GFI (green box), which contains one or more alternatively spliced exons together with flanking intronic sequences, is surrounded by 25 bps long attB1 and attB2 sites (dark blue boxes). Important sequences of the pSpliceExpress vector are those of the ccdB gene (red box), the chloramphenicol resistance gene (white box), the attP1 and attP2 sites (orange boxes), the Amp^R^ gene (light pink box), and the rat insulin 2 exons 2 and 3 (burgundy boxes). The resulting Expression clone contains the sequences of the attL1 and attL2 sites (orange and dark blue circles) and the GFI. The ccdB gene and the chloramphenicol resistance gene segments flanked by attR1 and attR2 sites (dark blue and orange circles) form the by-product of the BP reaction. (**B**) The LR reaction is mediated by Int, IHF, and Xis and leads to the cloning of the GFI into the pDESTsplice vector. Important sequence parts of the Entry clone are the GFI flanked by the attL1 and attL2 sites and an antibiotic resistance gene (e.g., for kanamycin resistance, purple box). The pDESTsplice vector principally contains the same sequence elements as the pSpliceExpress vector, except that pDESTsplice contains the attR sites instead of the attP sites. The resulting Expression clone contains the GFI flanked by the attB sites. The by-product is a vector containing the ccdB gene sequence and the chloramphenicol resistance gene sequence flanked by the attP sites. The sequences of attB1/B2, attP1/P2, attL1/L2, and attR1/R2 represent the forward strand only and are displayed in the same colors as in the schemes above. Amp^R^: ampicillin resistance, att: attachment, Cm^R^: chloramphenicol resistance, GFI: genomic fragment of interest, IHF: integration host factor proteins, Int: integrase, Xis: excisionase.

The BP reaction is based on the integration mechanism of phage λ (Figure 2A). It occurs between the mutated attachment sites derived from the *bacterial* chromosome (attB1/B2), which flank the GFI, and the attachment sites taken from *phage* λ DNA (attP1/P2) in the pSpliceExpress vector. The attB sites can be added in the GFI during PCR by using appropriate primers. The mutations in the attachment sites ensure site-specific recombination of attB1 with attP1 and of attB2 with attP2. The BP reaction is mediated by the integrase (Int) and integration host factor (IHF) proteins, leading to the integration of the GFI in the pSpliceExpress vector. The resulting minigene construct (hereafter called Expression clone) contains attachment sites that are called attL1 and attL2, as they consist of the *left* attachment site of the phage and the right attachment site of the bacteria. The by-product, namely, a fragment of the sequence with the ccdB gene and the chloramphenicol resistance gene, is flanked by the attachment sites attR1 and attR2. The latter attachment sites are named after the *right* attachment site of the phage and the left attachment site of the bacteria.

The creation of an Expression clone with pDESTsplice relies on the phage λ excision mechanism via the LR reaction (Figure 2B). The LR reaction is mediated by Int, IHF, and an excisionase (Xis) and occurs between the attL sites, which flank the GFI within a Gateway donor vector, and the attR sites of the pDESTsplice destination vector. A Gateway donor vector, which contains the GFI, is called Entry clone. After site-specific recombination of the attL1 and attL2 sites of the Entry clone with the attR1 and attR2 sites of the pDESTsplice vector, the resulting Expression clone contains the attB1 and attB2 sites. The by-product of the LR reaction is the donor vector with a sequence fragment containing the ccdB gene and the chloramphenicol resistance gene flanked by attP1 and attP2 sites.

**Figure 3:**
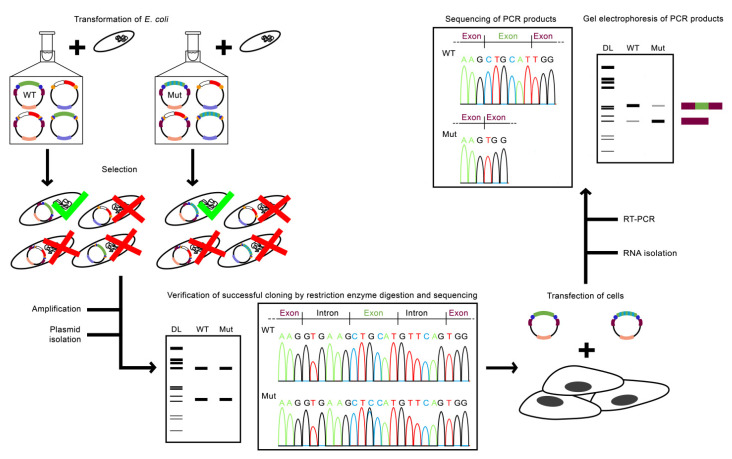
Typical workflow for assessing the impact of a genetic variant on splicing by minigene assay. Example Expression clones are shown, where two variants of a GFI are cloned into pDESTsplice. The variants of the Expression clone contain GFIs, which here represent two allelic variants of a SNP, one of them originating from a nucleotide substitution of G to C (WT: G, green box and Mut: C, green/light blue box). Constitutive splicing is expected for WT and alternative splicing for Mut. *E. coli* are transformed with a mixture containing the Expression clone, the by-product, pDESTsplice, and the Entry clone. For the components of the different vectors, the same color pattern as in Figure 2 was used. Bacteria containing WT and Mut are efficiently selected due to the ampicillin resistance gene and the ccdB gene. The selection is illustrated by green ticks (bacteria survive) and red crosses (bacteria cannot survive). The plasmid DNA is amplified and isolated. The verification of WT and Mut can be performed by restriction enzyme digestion and sequencing of extracted DNA bands. WT and Mut Expression clones are used to transfect cells. The transfected cells are incubated usually for 24 to 48 h before the RNA is isolated. The RNA is used for RT-PCR. The PCR products of WT and Mut are confirmed by gel electrophoresis and by sequencing. In the illustrated example, the Mut genotype leads to preferential skipping of the investigated exon. DL: DNA ladder, GFI: genomic fragment of interest, Mut: mutant, RT-PCR: reverse transcriptase polymerase chain reaction, WT: wild-type.

Next, bacteria are transformed with the minigene construct. In Figure 3, the general workflow of using a minigene assay to assess the impact of a SNP on pre-mRNA splicing with the pDESTsplice vector is shown as an example. Two allelic variants of a SNP can be obtained by site-specific mutagenesis within the minigene construct [37]. Both reactions, BP and LR, lead to a mixture of four different pieces of DNA: the Expression clone, the by-product, and the input sequence fragments and vectors that have not been recombined. The selection of the bacteria carrying the minigene construct is based on the ampicillin resistance (Amp^R^) gene and the ccdB gene. First, only bacteria that either contain the Expression clone or the original pDESTsplice or pSpliceExpress vector with the Amp^R^ gene can grow on ampicillin-containing medium. Second, due to the toxic effect of the ccdB gene product, the survival of bacteria that contain the original pDESTsplice or pSpliceExpress vector is affected. By this means, bacteria containing the Expression clone can be efficiently selected. *E. coli* strains without the F’ episome, such as TOP10, are recommended for the transformation, since the F’ episome contains the ccdA gene, which encodes an antitoxin that binds to ccdB, resulting in a conformational change of the toxin that prevents its binding to the gyrase and thus the desired negative selection. The successful cloning of the Expression clone can be examined by restriction digestion and Sanger sequencing.

The purified plasmid DNA, which constitutes the Expression clone, is used to transiently transfect cells. Cell lines, for which a high transfection efficiency can be achieved and which are therefore often used in minigene experiments, are HeLa and HEK293 cells (both human) as well as COS cells (African green monkey) [30]. Total RNA is usually isolated from transfected cells 24–48 h post-transfection. The detection of minigene RNA molecules, which have been transcribed in the cells, is typically performed by RT-PCR using primers that bind to the rat insulin exons flanking the ASE of interest, followed by gel electrophoresis and by Sanger sequencing of the PCR products after cutting out the specific bands from the gel.

There are some characteristics and pitfalls that should be considered when working with minigene assays in general and specifically when using Gateway cloning-based vectors such as pDESTsplice or pSpliceExpress. In general, the assay results must be considered with caution, as artificial effects may occur. In the minigene assay, only a specific part of a gene is examined, but the splicing of the exon of interest may be dependent on the proper splicing of other exons of the gene, which are not included in the Expression clone. It is also possible that important splicing factors are not sufficiently present in the cell line used.

By using the Gateway technology, one can generate Expression clones for the splicing reporter minigene assay within one week, and no restriction enzyme digestion is needed [4,38]. However, like the “scar” created by restriction enzymes, the attachment sites needed for Gateway cloning may also have the potential to slightly interfere with the experiment. As an advantage, the specific attachment sites for the Gateway cloning ensure directional cloning. GFI up to 4000 bps can be efficiently cloned into pDESTsplice or pSpliceExpress [4]. For larger GFI, the cloning efficiency decreases, but cloning into the vectors is still possible. A further aspect is that different Gateway donor vectors as well as ready-to-use mixes that contain the needed enzymes for the BP and LR reaction are commercially available.

An important step of the minigene assay is the transient transfection of a cell culture. Since expression of the introduced foreign gene construct only occurs over a certain period of time, dilution, or loss of the plasmids in the cells has to be considered. RNA isolation from the cells is usually performed up to 3 days after transfection, but the experimental result may still vary depending on the specific time point of cell harvest. Positive and negative controls are helpful to support the results and to interpret unexpected outcomes. For example, a minigene construct described in the literature could be used as a positive control for the assay workflow [4]. In cases where the minigene RNA products contain an open reading frame, it is important to consider the nonsense-mediated decay pathway. To prevent mRNA degradation by nonsense-mediated decay, in the experiment, one can use low concentrations of protein synthesis inhibitors, such as puromycin, added a few hours before RNA isolation [37].

The pDESTsplice and pSpliceExpress vectors combine the well-established features and advantages of the minigene assay with those of the Gateway cloning system. This optimization step makes these vector systems convenient for splicing reporter minigene assays. A further development of the splicing reporter minigene assay is the massively parallel reporter assay. Thus far, we are not aware that pDESTsplice and pSpliceExpress have been adapted for this approach. However, the use of the two vector systems for such application is conceivable. Massively parallel reporter assays allow for the simultaneous analysis of the functional impact of many different genetic variants on the splicing pattern [3]. They are based on the principle that a variety of sequences are cloned in reporter constructs [39,40]. The resulting plasmid library, which can consist of more than 2 million minigene constructs [40], is then used for the co-transfection of cells. Subsequently, associations between genetic variants and alternative splicing are detected using NGS for sequencing the DNA and cDNA libraries as well as complex bioinformatic pipelines.

## 6. Studies Using the pDESTsplice and pSpliceExpress Vectors

Both pDESTsplice and pSpliceExpress can be obtained from the non-profit plasmid repository Addgene (#32484 and #32485) for research purposes. This opportunity offers scientists the possibility to adapt the elaborated minigene systems in their own study. Depending on the objectives of the study, different experimental settings and laboratory protocols can be used. To give an overview of the potential applications of the pDESTsplice and pSpliceExpress vectors, we have compiled 25 published studies [41,42,43,44,45,46,47,48,49,50,51,52,53,54,55,56,57,58,59,60,61,62,63,64,65], in which these minigene systems were used (Table 1 and Table 2). In most of the works, the usage of pSpliceExpress has been described (*n* = 18). In five studies, the pDESTsplice system was used [43,49,50,53,65], and in two studies, pSpliceExpress derivatives were employed [57,59].

Minigene assays can be used to investigate splicing events in the context of various research topics. In the selected studies, the two vector systems were used to assess ASE in relation to diseases (*n* = 20) and to examine physiological mechanisms that regulate alternative splicing (*n* = 5).

The use of the minigene assay enables the analysis of different types of ASE. In the 25 studies, four different types of ASE were examined. Exon skipping was investigated 17 times, intron retention 9 times, and alternative splice sites and cryptic splice sites were investigated 3 times each. In two studies, the types of ASE were not specified [57,65]. ASE were studied in a total of 51 different gene transcripts. The splicing pattern of the CFTR mRNA was the only one that was analyzed in two reports [43,59]. The examined exons of all investigated genes are given in Table 2.

Alternative splicing can be influenced by various means. In most of the studies, the causal impact of genetic variants on ASE were analyzed (*n* = 19). Details of the tested genetic variants are listed in Table 2. In 3 of the 25 studies, the importance of RBPs was explored in co-transfection experiments. Sumanasekera et al. examined the influence of the sphingolipid signaling molecule C6 pyridinium ceramide on the regulation of splicing by protein phosphatase-1 [42], while Listerman et al. studied the regulatory role of SRSF11, hnRNPH2, and hnRNPL on exon 7/8 skipping of the telomerase reverse transcriptase gene transcript [44], and Xiao et al. assessed how the knockdown of *trans*-acting RBPs (YTHDC1, SRSF3, and SRSF10) affects the pre-mRNA splicing of ZNF638 [47]. Kishore et al. explored whether processed snoRNAs are involved in the selection of alternative splice sites by interfering with splicing regulatory proteins [41]. An altered splicing regulation through RNA modification was evaluated in two studies. Those modifications were implemented by altering RNA methylation either by creating knockout cells for the m^6^A mRNA demethylase FTO [49] or by introducing mutations in the m^6^A sequence motif [52].

Different sources can be used to obtain GFI of various lengths. In most of the 25 studies, genomic DNA was used to obtain the desired GFI (*n* = 18). In 2 of these 18 studies, the DNA was isolated from HeLa cells [44,47]. Payer et al. derived their inserts from genomic DNA and by gene synthesis [54]. Scott et al., Bartosovic et al., and Tang et al. also used synthetically produced sequences [43,49,65], while Kishore et al., Sumanasekera et al., and Chase et al. utilized bacterial artificial chromosomes to derive the wanted GFIs [41,42,60]. The size of the investigated GFIs in the 25 studies ranged from only 150 bps up to 7500 bps.

In order to examine genotype-dependent alterations in the splicing pattern, GFI that represent the original and the altered variant are required. In the selected studies, for the creation of different variants, mainly site-directed mutagenesis was performed (*n* = 9) or genomic DNA from subjects with different phenotypes was used (*n* = 9). Xiao et al., Bartosovic et al., and Wang et al. employed mutagenesis to introduce artificial mutations in RBP-binding regions or m^6^A modification sites [47,49,52]. For the DNA fragments used by Payer et al., Scott et al., and Tang et al., the different genetic variants were simply produced as part of the gene synthesis [43,54,65]. Payer et al. investigated minigene constructs that contained scrambled Alu sequences or randomized sequences of the same length as the polymorphic Alu element under scrutiny [54].

To create an Expression clone, the GFI must be cloned into pDESTsplice or pSpliceExpress. Gateway cloning with the pSpliceExpress vector relies on the BP reaction and thus no Gateway donor vector is required. However, Rittore et al. cloned some PCR fragments first into the TOPO-TA cloning vector and then into pSpliceExpress using restriction enzymes [45]. Since the Gateway cloning of the GFI into the pDESTsplice vector is based on the LR reaction, most investigators employ an Entry clone with the desired GFI. In the five studies in which the pDESTsplice vector was used, three compatible donor vectors were prepared, namely, the TOPO-TA cloning plasmid pCR™8, the pDONR221 vector, and the pENTR/D-TOPO vector [43,50,53,65]. Bartosovic et al. did not specify which vector they used [49].

As pDESTsplice and pSpliceExpress were designed for the application of Gateway cloning, this method was used for the generation of the minigene constructs in most studies (*n* = 18). However, in six studies, other cloning methods, namely, Gibson assembly, NEBuilder^®^, or restriction enzyme-based cloning, were used to generate the Expression clones (see Table 2). Payer et al. used two cloning procedures for their Expression clone generation, the Gateway cloning, and the Gibson assembly [54].

Verifying the successful cloning is important to ensure that the desired Expression clone and not a possible by-product is selected for the subsequent steps of the splicing reporter minigene assay. The examination of cloning success has been mentioned in 18 studies. In most of these studies, the assumed Expression clone was sequenced (*n* = 10) or a combination of sequencing and control cleavage with restriction enzymes was performed (*n* = 7). Varga et al. checked their Expression clones by colony PCR in addition to sequencing [56].

The next step in the splicing reporter minigene assay workflow is the transfection of cells with the Expression clone. The majority of cell lines used in the studies were human transformed embryonic kidney HEK293 cells (*n* = 16) or cervix carcinoma HeLa cells (*n* = 7). Other cell lines used were lung adenocarcinoma A549 cells (*n* = 1), cystic fibrosis cell line IB3-1 (*n* = 1), erythroleukemic K562 cells (*n* = 2), neuroblastoma Neuro2A cells (*n* = 1), and colon adenocarcinoma SW480 cells (*n* = 1). In four studies, two cell lines were used to replicate the results and to verify that the ASE is independent of the cell type: Cavill et al. used K563 and A549 cells [50], Chase et al. used HEK293 and HeLa cells [60], Rittore et al. HEK293 and SW480 cells [45], and Scott et al. K562 and IB3-1 cells [43]. After transient transfection, the cells are incubated for a certain time before they are harvested to isolate the RNA. The time point of RNA isolation that was chosen in the studies was usually 24 h (*n* = 9) or 48 h after transfection (*n* = 12). In the study by Beaman et al., the RNA was isolated 20 h after transfection [57]. In the remaining studies, it has not been reported at which specific time point after transfection the cells were harvested to isolate RNA [41,42,45].

RT-PCR followed by gel electrophoresis, often in combination with sequencing of the PCR products, is a typical workflow for the detection of minigene RNA molecules transcribed in the cells. These procedures were used in 21 of the 25 studies. During RT-PCR, PCR-generated DNA fragments of different lengths are produced for the different RNA isoforms. The differently sized PCR products are then visualized in a gel. However, other procedures were also applied. Legendre et al. performed a nested RT-PCR within the region of the lariat structure to determine the branch point [51]. After PCR, the amplicons were differentiated in size by fluorescent capillary electrophoresis instead of a gel electrophoresis [51]. In fluorescence capillary electrophoresis, DNA molecules are separated by passing them through a small capillary tube filled with a conductive buffer. The DNA is detected by fluorescent labeling using a DNA-binding dye. Instead of RT-PCR with endpoint analysis, Listerman et al., Rittore et al., and Carvill et al. used real-time RT-qPCR to quantify the relative expression levels of minigene RNA isoforms more accurately [44,45,50]. To assess the protein expression of splicing factors, Listerman et al. and Xiao et al. additionally performed Western blots [44,47].

## 7. Summary

ASE can be causally linked to the genotype of *cis*-regulatory genetic variants, such as SNPs or polymorphic Alu sequences, and to the presence of *trans*-acting splicing regulatory factors and pre-RNA modifications, such as methylation. The possibility to investigate ASE under defined experimental conditions, in which only one factor is perturbed, allows us to study the determinants of alternative splicing. The minigene assay approach is the method of choice for investigating the regulation of specific ASE, such as exon skipping, intron retention, and alternative splice site usage. The Gateway system-based vectors pDESTsplice and pSpliceExpress facilitate the fast generation of minigene constructs without the need for restriction enzyme target sequences. The size of the inserted genomic sequences can vary from a few 100 bps to several 1000 bps. The site-specific recombination of the Gateway cloning system ensures that a GFI is cloned directionally into pDESTsplice and pSpliceExpress, respectively. The dual selection pressure via the Amp^R^ gene and the toxic ccdB gene allows for efficient generation of desired Expression clones. For the detection of minigene RNA molecules, well-established procedures such as RT-PCR assays and sequencing of the PCR products can be performed. Further application of these minigene assays is expected to yield a better understanding of the regulation of splicing isoforms and may help to resolve functional implications of genetic variation underlying diseases.

## Figures and Tables

**Table 1:** Studies that explored alternative splicing by using pDESTsplice or pSpliceExpress.

Reference	Vector	Issue	Context	Study Topic	ASE	Special Features
Abdulhay et al., 2019 [55]	pSpliceExpress	Disease-related	Hematopoiesis	Genetic variants	Intron retention	Investigations on an intronic mutation disrupting the activity of snRNP U2
Alaa el Din et al., 2015 [46]	pSpliceExpress	Disease-related	Hereditary hemorrhagic telangiectasia syndrome	Genetic variants	Alt. splice site, Intron retention	Examination of the pathogenicity of genetic variants and their influence on splicing
Bartosovic et al., 2017 [49]	pDESTsplice	Physiological role	RNA modification via FTO demethylase	RNA modification	Exon skipping	Comparison of splicing regulation in FTO knock-out cells vs. wildtype cells
Beaman et al., 2019 [57]	derivate of pSpliceExpress	Disease-related	Urinary bladder disease	Genetic variants	N/A	Case report
Cao et al., 2020 [62]	pSpliceExpress	Physiological role	pMEIs	Genetic variants	Exon skipping	Experimental validation of pMEI sQTLs based on data from the GTEx project
Carvill et al., 2018 [50]	pDESTsplice	Disease-related	Dravet syndrome and related genetic epilepsies	Genetic variants	Intron retention	Analysis of a new genetic variant identified by genome sequencing in a patient
Chase et al., 2020 [60]	pSpliceExpress	Disease-related	Myeloid neoplasms	Genetic variants	Exon skipping	Effect of mutations on methylation activity and the splicing process
Dupont et al., 2019 [58]	pSpliceExpress	Disease-related	Diseases linked to cilium	Genetic variants	Exon skipping	Comparison of IFT52 mutations in fetuses with distinct phenotypes
Ellingford et al., 2019 [59]	derivate of pSpliceExpress	Disease-related	Rare monogenic disorders	Genetic variants	Alt. splice site, Cryptic splice site, Exon skipping, Intron retention	Experimental set-up to examine accuracy of in silico variant prioritization strategies
Kishore et al., 2010 [41]	pSpliceExpress	Disease-related	Prader–Willi syndrome	Splicing regulators	Exon skipping, Intron retention	Cotransfection of target minigenes and SNORD 115 expression constructs
Knapp et al., 2020 [63]	pSpliceExpress	Disease-related	Meier–Gorlin syndrome	Genetic variants	Intron retention	Identification of novel genetic variants in genes that cause disease
Legendre et al., 2018 [51]	pSpliceExpress	Disease-related	CHARGE syndrome	Genetic variants	Intron retention	Branch point analyses
Listerman et al., 2013 [44]	pSpliceExpress	Disease-related	Cancer biology	Splicing regulators	Exon skipping	SRSF11, hnRNPH2 and hnRNPL regulate TERT exon 7/8 skipping
Mattison et al., 2018 [53]	pDESTsplice	Disease-related	Epilepsy	Genetic variants	Exon skipping	Splicing studies on genetic variants discovered in patients
Mutai et al., 2020 [64]	pSpliceExpress	Disease-related	Hereditary hearing loss	Genetic variants	Exon skipping	Combination of minigene assays and functional analyses in cochlear tissues
Payer et al., 2019 [54]	pSpliceExpress	Physiological role	Alu polymorphisms	Genetic variants	Exon skipping	Influence of Alu element polymorphisms on splicing
Rittore et al., 2014 [45]	pSpliceExpress	Disease-related	Inflammatory diseases	Genetic variants	Exon skipping	Assessment of combinatorial effects of SNPs
**Reference**	**Vector**	**Issue**	**Context**	**Study Topic**	**ASE**	**Special Features**
Scott et al., 2012 [43]	pDESTsplice	Disease-related	Cystic fibrosis	Genetic variants	Cryptic splice site, Exon skipping	Selection of genetic variants for experimental testing via bioinformatic tools
Starokadomskyy et al., 2016 [48]	pSpliceExpress	Disease-related	X-linked late pigmentary disorder	Genetic variants	Intron retention	Investigations on an intronic mutation causing a rare X-chromosomal disease
Sumanasekera et al., 2012 [42]	pSpliceExpress	Disease-related	Ceramide-mediated splicing, Cancer drug	Splicing regulators	Alt. splice site, Exon skipping	Influence of C6 pyridinium ceramide on splicing
Tang et al., 2020 [65]	pDESTsplice	Disease-related	Alzheimer’s disease	Genetic variants	N/A	Investigations of genotype-dependent splicing efficiencies
Thomas et al., 2020 [61]	pSpliceExpress	Disease-related	Mandibulofacial dysostosis Guion–Almeida type	Genetic variants	Cryptic splice site, Exon skipping, Intron retention	Investigations on pathogenic variants altering splicing of the human EFTUD2 gene and the yeast homolog SNU114
Varga et al., 2019 [56]	pSpliceExpress	Disease-related	Autosomal dominant sensorineural hearing loss	Genetic variants	Exon skipping	Case report
Wang et al., 2018 [52]	pSpliceExpress	Physiological role	Mammalian cerebellar development	RNA modification	Exon skipping	Aberrant splicing due to METTL3-mediated m^6^A modification
Xiao et al., 2016 [47]	pSpliceExpress	Physiological role	Splicing regulatory factors, RNA-binding proteins	Splicing regulators	Exon skipping	Splicing regulation of ZNF638 upon knockdown of YTHDC1, SRSF3 or SRSF10

We compiled 25 articles (sorted by author name) that described a minigene splicing assay on the basis of the pDESTsplice or pSpliceExpress vector systems. The physiological role of splicing variants or their relation to diseases was investigated. The influences on splicing by genetic variants, RNA modifications or splicing regulatory factors were examined in the 25 studies. The investigated alternative splicing patterns can be distinguished into 4 different types of ASE: alternative splice sites, cryptic splice sites, exon skipping, and intron retention. The specific research context and special features of the individual studies were also recorded in the table. Alt.: alternative, ASE: alternative splicing events, N/A: not available, pMEIs: polymorphic mobile element insertions, SNP: single-nucleotide polymorphism, sQTLs: splicing quantitative trait loci.

**Table 2:** Experimental setup for the minigene assays applied in the 25 studies.

Reference	Genes	Exons	Genetic Variants	Source (Size)	Variant Creation	Donor Vector	Cloning	Construct Verification	Cells	RNA Iso.	Detection Procedures
Abdulhay et al., 2019 [55]	GATA1	Exon 5–6	chrX:48652176C>T (hg19)	DNA (1335 bp *)	Control & patient	/	RE	N/A	HEK293T	48 h	RT-PCR, Gel electrophoresis, Sequencing (PCR product)
Alaa el Din et al., 2015 [46]	ACVRL1	Exon 6, Exon 7, Exon 9	c.733A>G, c.1249A>T, c.1048+5G>A	DNA (≈500–700 bp)	N/A	/	Gateway	RE digestion, Sequencing (plasmid)	HeLa	48 h	RT-PCR, Gel electrophoresis, Sequencing (PCR product)
Bartosovic et al., 2017 [49]	BRD8	Exon 20–21	/	Gene synthesis (1202 bp *)	Site-directed mutagenesis	N/A	Gibson Assembly	N/A	HEK293T	24 h	RT-PCR, Gel electrophoresis
Beaman et al., 2019 [57]	CHRM3	Exon 7 *	c.352G>A	DNA (840 bp)	Control & patient	/	NEBuilder^®^	Sequencing (plasmid)	HEK293	20 h	RT-PCR, Gel electrophoresis, Sequencing (PCR product)
Cao et al., 2020 [62]	SS18L1, CAP1, IFT122	Exon 2–3 (SS18L1) *, Exon 2 (CAP1) *, Exon 8–9 (IFT122) *	pMEIs	DNA (≈2800–5500 bp *)	N/A	/	Gateway	Sequencing (plasmid)	HEK293T	24 h	RT-PCR, Gel electrophoresis
Carvill et al., 2018 [50]	SCN1A	Exon 20–21	chr2:166864064G>A, chr2:166864057_166864061del, chr2:166863778C>G, chr2:166863774C>T, chr2:166863726G>A (hg19)	DNA (≈7500 bp)	Site-directed mutagenesis	pDONR221	Gateway	Sequencing (plasmid)	K562, A549	24 h	RT-qPCR
Chase et al., 2020 [60]	EZH2	Exon 8	Y244D, E249K, L252V, A255T, R288Q, H297R, R298L	BAC-derived PCR fragment (1543 bp *)	Site-directed mutagenesis	/	Gateway	N/A	HEK293F, HeLa	48 h	RT-PCR, Gel electrophoresis
Dupont et al., 2019 [58]	IFT52	Exon 8	c.695–699delinsCA	DNA (464 bp *)	Control & patient	/	Gateway	N/A	HEK293T	48 h	RT-PCR, Gel electrophoresis
Ellingford et al., 2019 [59]	ABCA4, GUCY2D, PDE6B, MERTK, SCN2A, ABHD12, CRYBA1, DNAH11, CFTR, RPGR, MYBPC3, TRPM1	N/A	NM_000350.2:c.5584+6T>C, NM_000180.3:c.3043+5G>A, NM_000283.3:c.2130-15G>A, NM_006343.2:c.2486+6T>A, NM_001040142.1:c.2919+3A>G, NM_015600.4:c.867+5G>A, NM_005208.4:c.213C>T, NM_001277115.1:c.6547-963G>A, NM_000492.3:c.3874-4522A>G, NM_001034853.1:c.247G>T, NM_001034853.1:c.1754-3G>C, NM_000256.3:c.1224-21A>G, NM_002420.5:c.899+29G>A	DNA (N/A)	Control & patient	/	NEBuilder^®^	Sequencing (plasmid)	HEK293	48 h	RT-PCR, Gel electrophoresis, Sequencing (PCR product)
**Reference**	**Genes**	**Exons**	**Genetic Variants**	**Source (Size)**	**Variant Creation**	**Donor Vector**	**Cloning**	**Construct Verification**	**Cells**	**RNA Iso.**	**Detection Procedures**
Kishore et al., 2010 [41]	DPM2, TAF1, RALGPS1, PBRM1, CRHR1	N/A	/	BAC-derived PCR fragments (N/A)	/	/	Gateway	RE digestion, Sequencing (plasmid)	Neuro2A	N/A	RT-PCR, Gel electrophoresis
Knapp et al., 2020 [63]	DONSON	Exon 3–5	c.607-36G>A	DNA (3130bp *)	Control & patient	/	Gateway	Sequencing (plasmid)	HeLa	24 h	RT-PCR, Gel electrophoresis, Sequencing (PCR product)
Legendre et al., 2018 [51]	CHD7	Exon 26	rs398124321, rs1131690787, rs794727423, rs199981784	DNA (566bp)	Site-directed mutagenesis	/	Gateway	Sequencing (plasmid)	HeLa	48 h	RT-PCR, Fluorescent capillary electrophoresis, Nested lariat RT-PCR, Sequencing (PCR product)
Listerman et al., 2013 [44]	TERT	Exon 5–9	/	DNA from HeLa (N/A)	/	/	Gateway	RE digestion, Sequencing (plasmid)	HEK293T	48 h	RT-qPCR
Mattison et al., 2018 [53]	SLC6A1	Exon 8–10	c.850-2A>G	DNA (1450bp)	Site-directed mutagenesis	pENTR/D- TOPO	Gateway	RE digestion, Sequencing (plasmid)	HEK293	24 h	RT-PCR, Gel electrophoresis, Sequencing (PCR product)
Mutai et al., 2020 [64]	SLC12A2	Exon 21–22	c.2930-2A*>*G	DNA (2507bp)	Control & patient	/	Gateway	Sequencing (plasmid)	HEK293T	48 h	RT-PCR, Gel electrophoresis, Sequencing (PCR product)
Payer et al., 2019 [54]	NUP160, CCDC110, BPIFC, SLC2A9, CD58	Exon 33 (NUP160), Exon 5 (CCDC110), Exon 10–11 (BPIFC), Exon 2 (SLC2A9), Exon 3 (CD58)	AluYh3a3 (ALU_umary_ALU_8566), AluY (ALU_umary_ALU_4001), AluYa5 (ALU_umary_ALU_12481), AluYi6 (RIP-041), AluY (DEL_pindel_1315)	DNA, Gene synthesis (≈1000–2400bp *)	Control & patient, Gene synthesis	/	Gateway, Gibson Assembly	Sequencing (plasmid)	HEK293T	24 h	RT-PCR, Gel electrophoresis
Rittore et al., 2014 [45]	TNFRSF1A	Exon 1–4, Exon 1–2, Exon 2–4	rs1800692, rs4149570, rs767455	DNA (≈800–1600bp)	Site-directed mutagenesis	TOPO-TA	RE	N/A	HEK293T, SW480	N/A	RT-qPCR
Scott et al., 2012 [43]	CFTR	Exon 6, Exon 8, Exon 15, Exon 22	rs35033453, rs1800083, rs1800084, rs1800105, rs1800122	Gene synthesis (≈250–400bp)	Gene synthesis	TOPO-TA (pCR™8)	Gateway	RE digestion, Sequencing (plasmid)	K562, IB3-1	24 h	RT-PCR, Gel electrophoresis
Starokadomskyy et al., 2016 [48]	POLA1	Exon 13–14	NC_000023.10:g.24744696A>G	DNA (N/A)	Control & patient	/	Gateway	Sequencing (plasmid)	HEK293	48 h	RT-PCR, Gel electrophoresis, Sequencing (PCR product)
**Reference**	**Genes**	**Exons**	**Genetic Variants**	**Source (Size)**	**Variant Creation**	**Donor Vector**	**Cloning**	**Construct Verification**	**Cells**	**RNA Iso.**	**Detection Procedures**
Sumanasekera et al., 2012 [42]	DBF4B, MYO18A ^b^, POLB, MAPT, SYK	Exon 10 (DBF4B) *, Exon 39–41 (MYO18A) *, Exon 1–2 (POLB) *, N/A (SYK, MAPT)	/	BAC-derived PCR fragments (≈2200–4400bp *)	/	/	Gateway	RE digestion, Sequencing (plasmid)	HEK293	N/A	RT-PCR, Gel electrophoresis
Tang et al., 2020 [65]	CDH23, SLC9A3R1	Exon 50 (CDH23), Exon 3 (SLC9A3R1)	rs56013867, rs41282067	Gene synthesis (≈150–230 bp)	Gene synthesis	pENTR/D- TOPO	Gateway	RE digestion, Sequencing (plasmid)	HEK293	24 h	RT-PCR, Gel electrophoresis, Sequencing (PCR product)
Thomas et al., 2020 [61]	EFTUD2	Exon 5, Exon 9, Exon 10, Exon 13 Exon 15, Exon 16, Exon 18, Exon 19, Exon 20, Exon 23, Exon 25, Exon 26, Exon 27	c.428C>T, c.620G>A, c.623A>G, c.670G>A, c.670G>C, c.784C>T c.857A>G, c.1149G>C, c.1306C>G, c.1426T>C, c.1496G>A, c.1732C>T, c.1860G>C, c.1860G>T, c.1910T>G, c.2033C>A, c.2305G>C, c.2332C>T, c.2467G>A, c.2485G>A, c.2566C>T, c.2813G>A	DNA (N/A)	Site-directed mutagenesis	/	Gibson Assembly	Sequencing (plasmid)	HEK293	48 h	RT-PCR, Gel electrophoresis, Sequencing (PCR product)
Varga et al., 2019 [56]	EYA4	Exon 10	c.804G>C	DNA (N/A)	Patients	/	Gateway	Colony PCR, Sequencing (plasmid)	HeLa	24 h	RT-PCR, Gel electrophoresis, Sequencing (PCR product)
Wang et al., 2018 [52]	Lrp8 ^a^, Grin1 ^a^	Exon 19 (Lrp8), Exon 21 (Grin1)	/	N/A (≈1000–1400bp *)	Site-directed mutagenesis	/	N/A	N/A	HeLa	48 h	RT-PCR, Gel electrophoresis
Xiao et al., 2016 [47]	ZNF638	Exon 2	/	DNA from HeLa (≈2500bp)	Site-directed mutagenesis	/	Gateway	N/A	HeLa	48 h	RT-PCR, Gel electrophoresis

Alternative splicing events of 51 different genes were examined. The Gateway vectors pDESTsplice and pSpliceExpress and the used donor vectors for the Entry clone creation are given in the table. If no information from the study was available, it was marked as N/A in the corresponding field, and if a procedure was not used in the study, a slash was noted (/). In cases where genetic variants were examined, they were recorded in the table. The investigated exons and the source of the genomic sequence of interest, namely, genomic DNA from donors, DNA extracted from cell lines, gene synthesis fragments, or BAC-derived PCR fragments, are recorded. In addition, the fragment size is given. If only one exon was examined, the exact size was noted; however, if several exons have been examined, the range of the approximate insert size is given. In cases where the examined exons or the insert sizes have not been described but could be calculated on the basis of the given primers, the specifications in the table are marked with an asterisk (*). We also recorded how the investigated variants were created, which cloning procedure was applied, and how the minigene constructs were verified. Furthermore, we noted the cell lines, which were transiently transfected with the minigene constructs and checked how long they were incubated before the RNA was isolated. Procedures used to detect the transcribed minigene RNA molecules were RT-PCR followed by gel electrophoresis or fluorescent capillary electrophoresis, nested lariat RT-PCR, RT-qPCR, and (Sanger) sequencing. ^a^ Investigations were performed on genes from mice. ^b^ According to the current human genome assembly of the UCSC Genome Browser (Dec. 2013, hg38), the investigated splicing event concerns the MYO18A gene and not the TIAF1 gene as annotated in previous genome assemblies. BAC: bacterial artificial chromosome, N/A: not available, RE: restriction enzyme, RNA iso: RNA isolation time point (post-transfection), RT-PCR: reverse transcriptase polymerase chain reaction, RT-qPCR: quantitative reverse transcription polymerase chain reaction.

## Abbreviations

Alt.	Alternative
Amp^R^	Ampicillin resistance
ASE	Alternative splicing events
att	Attachment site
BAC	Bacterial artificial chromosome
BBP	Branch point-binding protein
bps	Base pairs
ccd	Control of cell death
Cm^R^	Chloramphenicol resistance
ESE	Exonic splicing enhancer
ESS	Exonic splicing silencer
GFI	Genomic fragment of interest
hnRNP	Heterogeneous nuclear ribonucleoprotein
IHF	Integration host factor
Int	Integrase
ISE	Intronic splicing enhancer
ISS	Intronic splicing silencer
N/A	Not available
NGS	Next-generation sequencing
pMEIs	Polymorphic mobile element insertions
PPT	Polypyrimidine tract
RBP	RNA-binding protein
RE	Restriction enzyme
RT-PCR	Reverse transcriptase PCR
RT-qPCR	Quantitative reverse transcriptase PCR
snoRNA	Small nucleolar RNA
SNP	Single-nucleotide polymorphism
snRNP	Small nuclear ribonucleoprotein
SRSF	Serine/arginine-rich splicing factor
U2AF	U2 auxiliary factor
Xis	Excisionase
